# 

**Published:** 1911-05-15

**Authors:** 


### ANNOUNCEMENTS.

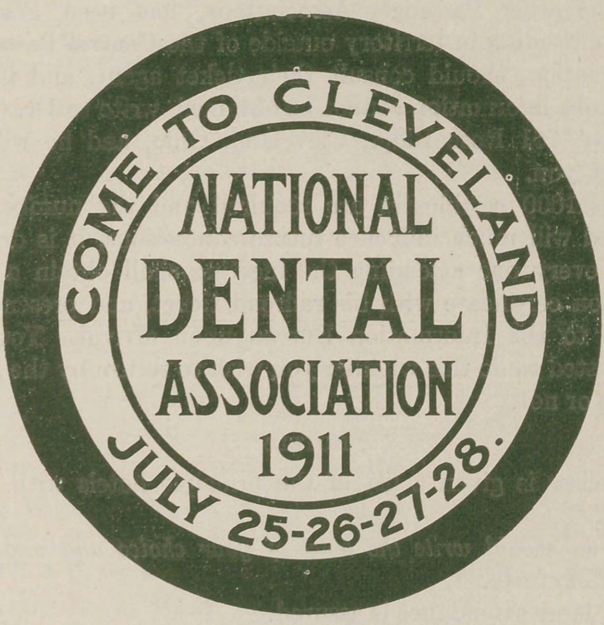

### COME TO CLEVELAND FOR THE NATIONAL DENTAL ASSOCIATION.

JULY 25-26-27-28, 1911.

PROGRAM:

A full literary program—approximately 300 clinics—unusually large and varied dealers' exhibit—an ideal convention city, and your presence required to further the great interests confronting the profession of to-day; viz: State and National reorganization, Oral Hygiene, National Journal, and gooc-fellowship.

RAILROAD RATES:

Since the advent of the 2 cent fare,. concessions have not been made for our National Meetings, but this year a concession of one and one-half fare (certificate plan) by the Central Passenger Association, with modifications by the other Passenger Associations, has been granted. Those residing in territory outside of the Central Passenger Association, should consult their ticket agent, and if satisfactory information cannot be obtained, write to Dr. G. D. Lovett, 761 Rose Bldg., Cleveland, Ohio, and he will instruct you.

As 1000 certificates are required and the number returned will much influence future concessions, it is desired that every one attending the meeting shall obtain a convention certificate with his railroad ticket, and present the same to the Information Bureau upon arrival. You are requested to do this whether you wish to return by the same route or not.

HOTELS:

Below is given a list of the principle hotels with their rates.

*You should write the hotel of your choice and make reservations early.*

A large attendance is assured.

EUROPEAN PLAN.

| | Single Without Bath | Single With Bath | Double Without Bath | Double With Bath |
|---|---|---|---|---|
| Hollenden Hotel, Superior and E. 6th. Headquarters for National Dental Association and National Faculties Association | $2.00 | $2.50<br>$3.00<br>$3.50<br>$4.00 | $3.00 | $4.00 and $5.00 |
| Colonial Hotel, Colonial Arcade and Prospect Avenue. Headquarters for National Association Dental Examiners | $2.50 | $3.00 and $4.00 | $4.00 and $4.50 | $5.00 and $5.50 |
| Hotel Euclid, Euclid Ave. and E. 14th. Headquarters for Clinicians | $1.50 and $2.00 | $2.00<br>$2.50<br>$3.00<br>$4.00 | $2.50 and $3.00 | $3.00<br>$3.50<br>$4.00<br>$5.00 |

| | Single Without Bath | Single With Bath | Double Without Bath | Double With Bath |
|---|---|---|---|---|
| Gilsey Hotel (Stag), East 9th Street | -----<br>----- | -----<br>----- | $2.00<br>$3.00 | $3 50<br>$4.00 |
| Tavistock & Wyandotte, Huron Road and East 9th St. | $1.00<br>$1.50 | $2.00<br>$3.00 | $2.00<br>$2.50 | $3.00<br>----- |
| Mooreland Hotel, 1600 Euclid Ave. | $1.00 | $1.50<br>$2.00 | $1.50 | $2.00<br>$3.00 |
| Fuller—Euclid Ave. and East 17th St. | $1.00 | $1.50 | $1.50 | ----- |
| Hawley House, St. Clair and West 3rd St. | $ .75<br>$1.00<br>$1.50 | ----- | ----- | ----- |
| American House, Superior and West 4th St. | $1.00<br>$1.50 | ----- | ----- | ----- |
| Kennard House, St. Clair and West 6t St. | $1.00<br>$1.50 | $2.00<br>$2.50 | $2.00<br>$2.50 | $3.50<br>$4.50 |

AMERICAN PLAN.

| | | | | |
|---|---|---|---|---|
| Colonial Hotel, "A Famous Cuisine" | $4.00<br>$4.50 | $4.50<br>$5.00 | $7.00<br>$8.00 | $8.00<br>$9.00 |
| Tavistock & Wyandotte | $2.00 | $2.50 | $4.00 | $5.00 |
| Forest City House, Public Square | $2.00<br>$2.50<br>$3.00 | $3.50 | --- | ----- |
| American House | $2.00<br>$3.00 | ----- | ----- | ----- |
| Kennard House | $2.00<br>$2.50 | $3.00<br>$3.50 | $4.00<br>$5.00 | $6.00<br>$7.00 |

MEMBERSHIP:

All members of State Associations are eligible for membership in the National Association on certificate signed by the Officers of his association.

Make this a part of your vacation trip, a royal time is assured you.

For Information Address—

Local Committee of Arrangements.

National Dental Association,

718 Schofield Bldg., Cleveland, Ohio.

### STATE SOCIETY MEETING.

### JUNE.

ARKANSAS STATE DENTAL ASSOCIATION. Pine Bluff. Three days: June 7th to 9th.

COLORADO STATE DENTAL ASSOCIATION. Boulder. Three days: June 29th to July 1st.

FLORIDA STATE DENTAL SOCIETY. Pensacola. Three days: June 20th to 22nd.

GEORGIA STATE DENTAL SOCIETY. Macon. June 8th.

MAINE, NEW HAMPSHIRE, AND VERMONT DENTAL SOCIETIES. Fabyan, N. H. Four days: June 27th to 30th.

MINNESOTA STATE DENTAL ASSOCIATION. Minneapolis. Two days: June 9th and 10th.

MISSOURI STATE DENTAL ASSOCIATION. Joplin. Three days: June 13th to 15th.

MONTANA STATE DENTAL SOCIETY. Helena. Two Days June 2nd and 3d.

NEW HAMPSHIRE, MAINE, AND VERMONT DENTAL SOCIETIES. Fabyan. Four days: June 27th to 30th.

NORTH CAROLINA STATE DENTAL SOCIETY. Morehead City. Four days: June 28th to July 1st.

OKLAHOMA STATE DENTAL ASSOCIATION. Enid. Three days: June 1st to 3d.

PENNSYLVANIA STATE DENTAL SOCIETY. Scranton. Three days: June 27th to 29th.

SOUTH CAROLINA STATE DENTAL SOCIETY. Columbia. Three days: June 20th to 22d.

SOUTHERN WISCONSIN DENTAL ASSOCIATION. Madison. Two days: June 1st and 2d.

VERMONT, MAINE, AND NEW HAMPSHIRE DENTAL SOCIETIES. Fabyan. Four days: June 27th to 30th.

VIRGINIA STATE DENTAL ASSOCIATION. Richmond. Three days: June 14th to 16th.

WASHINGTON STATE DENTAL SOCIETY. Tacoma. Three days: June 1st to 3d.

### EXAMINERS' MEETINGS.

Florida Board of Examiners. Pensacola. June 15th to 17th and 19th.

Illinois Board of Examiners. Chicago. June 5th.

Maryland Board of Examiners. Baltimore. May 24th and 25th.

Michigan Board of Examiners. Ann Arbor. June 19th to 24th.

Nebraska Dental Board. Lincoln. June 26th.

New Hampshire Board of Registration. Manchester. June 1st to 3rd.

New Jersey Board of Registration. Trenton. June 26th to 28th.

Ohio State Dental Board. Columbus. June 20th to 23d.

Texas Board of Examiners. Dallas. June 19th.

Washington Board of Examiners. Tacoma. May 25th.

Wisconsin Board of Examiners. Milwaukee. June 12th.—*Cosmas.*

### NATIONAL ASSOCIATION OF DENTAL FACULTIES.

The next meeting of the National Association of Dental Faculties will take place at the Hollenden Hotel, Cleveland, Ohio, Saturday July 22 at 10 A. M. The Executive Committee will meet at 9 A. M. the same day.

Anyone having business to transact with the committee will please present it at that time.

B. Holly Smith,
Chmn. Executive Committee.

### SOUTH DAKOTA BOARD OF DENTAL EXAMINERS.

The South Dakota State Board of Dental Examiners will hold its next meeting at Sioux Falls S. D., Tuesday July 11th, 1911, at 1.30 P. M., and continuing three days. All applications for examination, together with a fee of twenty-five dollars, must be in the hands of the Secretary by July 1st. Applicants who have not complied with the above will not be permitted to take the examination.

For futher information, blanks, etc., address,

ARIS L. REVELL, *Sec.*

Lead, S. D.

---

### THE MICHIGAN STATE BOARD OF DENTAL EXAMINERS.

The next regular meeting of the Michigan State Board of Dental Examiners will be held at the Dental Cellege Building at Ann Arbor during the whole of the week of June 19th, beginning on Monday at 8.00 A. M. All applications and fees should be in the hands of the secretary at least ten days before that date. For full information, address:

A. W. HAIDLE, *Sec.*
Negaunee, Mich.

---

### ARKANSAS STATE BOARD OF DENTAL EXAMINERS.

Next meeting of the State Board of Dental Examiners will be held June 5th and 6th, 1911. Pine Bluff, Ark.

All applicants are required to pass a theoretical examination, fee $15.00. Examination covers all branches taught in dental schools. No temporary license issued. No interchange with any state.

A. T. MCMILLIN, *Sec'y-Treas.*
Little Rock, Ark.
405 and 406 State Bank Bldg.

### PERSONAL.

Removal—Dr. F. E. Williams, formerly practicing orthodontia at Grand Rapids, has removed to Los Angeles California. Dr. J. Ward House, for many years an active working member of the profession in Michigan has been during the past winter recuperating his health in California. We are pleased to announce that he expects to soon be able to take up practice again, probably in California, we are sorry to loose so good a man. Dr. V. F. Godfrey a well known dentist of Alpena, Mich., died February 22d, 1911. Dr. Samuel S. Mummer of Big Rapids, died March 9th, 1911. Dr. Edward W. Brannigan, Prof. of Operative Dentistry in Tufts College, Boston, died April 1st 1911. He was a capable teacher and well known and much beloved by all who knew him.

### NEW JERSEY STATE DENTAL SOCIETY.

The Forty-first annual meeting of the New Jersey State Dental Society will convene at Asbury Park, N. J., July 19th, 20th and 21st, 1911, beginning on Wednesday the 19th, at 10 A. M.

The Casino with extensive floor alteration has been secured for the exhibits which will be the largest and most complete ever given by any State Society, and modern dental equipments and up-to-date instruments will be shown. Application at an early date is necessary to secure space. Dr. W. W. Hawke of Flemington, N. J., is Chairman of the Exhibit Committee. Hotel Brunswick will be the headquarters of the Society which is large and spacious and near the Casino. On account of the noise from the Ocean it will be necessary to have the business meeting and the papers in the large ball room of the Brunswick, the clinics in the Casine.

The rates at the Brunswick are as follows: Two persons in one room $3.50 per day one person in room $4.00 per day, rooms with bath $1.00 extra.

Programmes will be out the first of July and will upon request be sent to ethical practitioners from other States contemplating a visit to the meeting.

CHARLES A. MEEKER, D.D.S.
29 Fulton St., Newark, N. J.

### MICHIGAN STATE DENTAL SOCIETY ANNUAL MEETING.

The meeting at Grand Rapids, April 10th to 12th, was one of the best in the society's history. It was largely attended and the programme in all its parts was most instructive and satisfactory. If anything in the way of criticism was made it was that there were too many clinics being given in a short space of time . Lets have fewer clinics or else more time. The reorganization of the state was brought more nearly to completion. by the meeting of the Southwestern Society with the State Society and there seems now a probability of an early consolidation of all the State Societies The proposed dental law was discussed and by a strong majority vote it was decided to postpone its recommendation until the meeting of the next legislature. The Sosiety voted to contribute three hundred dollars to the Columbus monument to honor Dr. W. D. Miller and a Committee was appointed to raise by professional subscriptions five hundred dollars for the International Miller Memorial Fund. A spirited but good natured contest between the Western and the Eastern Dentists over the election of officers took place. The Western contingent winning out and electing Dr. J. H. Armstrong of Belding, as President; C. G. Bates of Durand, Vice-President; M. L. Ward of Ann Arbor, Secretary and J. W. Lyons of Jackson, Treasurer. All good hustlers assuring another year of progressive work and a good meeting next year, probably in Detroit.

### Dr. Haskell's Book.

Everyone in the dental profession knows Dr. L. P. Haskell. but everyone does not know that he has written a book. Dr. Haskell has devoted sixty-five years of his life to one department of prosthetic dentistry—artificial dentures. He has written the essence of his experience in this volume, and it is now for sale by a committee selected by Dr. Haskell to represent him. He does this because he has more taste for the art of dentistry then he has for business. In fact he has so little taste for business that he has lived to the age of eighty-five without thinking much about it . No man ought to be compelled to work after he is eighty-five, and so Dr. Haskell's friends have decided that this book should be made to support him comfortably for the rest of his days. It is a small volume of sixty-three pages, and the price is $2.00. There is a splendid portrait of Dr. Haskell in the frontis piece and this itself is worth the price of the book to the many friends in the profession who know and love him.

Fraternally yours,

Dr. C. N. Johnson, *Chairman*,
31 Washington St. Chicago, Ill.

Committee, Dr. T. W. Brophy,
Dr. J. A. Bullard.

Order through your dealer or directly through the C. L. Frame Dental Supply Co., 1301 Masonic Temple, Chicago.

### Memorial to Dr. Claude Martin.

The Dental Association of Lyon, France, has started a subscription to endow a suitable memorial to Dr. Claude Martin one of the most illustrious French dentists of modern times, and since his work has been international in benefits they make this appeal to Americans.

Claude Martin has made for himself an universal and rightly deserved reputation. Beginning with modest resources he gradually attained as high a renown and esteem as were ever held by a dentist in this or any other country. By the dissemination of his methods his fame has become widespread.

The whole list of his inventions and original ideas would be too long to enumerate here.—Starting in our profession as a mechanical dentist, he soon reached the climax of perfection in his art. He was then led to apply the principles of mechanical dentistry to facial restoration following upon surgical intervention.

The late Dr. Ollier, the eminent surgeon of Lyons, proclaimed Claude Martin as his master. He first proved the perfect tolerance of wounded tissue for a fixed prosthetical appliance, thus avoiding the unsightly contraction of the features; the platinum skeleton form for the nose over which flap operations were performed; the porcelain nose, his original form of obturators in the treatment of cleft palates and above all his master piece of art: an artificial larynx permitting the dumb to speak, these were but a few of his genial. inventions. Recently his treatment for the regulation of sunken and broken noses was most successful and was obtained by the extension of dental methods.

At a comparatively advanced age Claude Martin took his medical degree and finally was admitted to that learned body the Academiede Medecine of Paris. The mechanical dentist had become an *Academicien*, one of the highest honors ever bestowed on a dentist. He liberally imparted his knowledge and discoveries in various publications. He was laureate of the *Institut of France, and Chevalier* of the Legion of Honor.

To honor such a man is to honor ourselves. Any subscription should be sent to Dr. Caillon, No. 1 Victor Hugo, Lyon, France.

Signed:

M. J. QUINTERO, D.D.S. H. J. HARWOOD, D.D.S.

